# Supplementary material for: Chimpanzees (Pan troglodytes) display limited behavioural flexibility when faced with a changing foraging task requiring tool use
Source: PeerJ. 2018 Feb 19;6:e4366. doi: 10.7717/peerj.4366 (PMC5822838; doi:10.7717/peerj.4366)
Supplement: Article S1 [file peerj-06-4366-s001.docx]

Supplemental Article S1

Techniques involving the insertion of a hand into the tube (ie. *hand dip*) were classified as ‘Partially effective’, as insertion of the hand into the tube was made impossible by the width of the tube in the ‘Narrow Tube’ phase, though this technique could be successfully used in the ‘Wide Tube’ phases. The median success rate individuals achieved using *hand dip* dropped significantly at the introduction of the ‘Narrow Tube’ (‘Wide Tube’ Mdn = 0.96, ‘Narrow Tube’ Mdn = 0; W=28, Z=2.37, p=0.016). Techniques reliant upon the insertion of a plain, rigid stick into the tube were also classified as ‘Partially effective’, as the overhang of the task presentation window was such that these sticks could often no longer be inserted into the tube in the ‘Narrow Tube’ phase. Individual’s success rates using ‘*stick dip*’ dropped significantly after the introduction of the ‘Narrow Tube’ (‘Wide Tube’ Mdn = 0.65, ‘Narrow Tube’ Mdn = 0.42; W=42, Z=2.25, p=0.023). The technique ‘*stick drop*’ did not see a significant reduction in its success rate due to the introduction of the ‘Narrow Tube’, and is therefore categorized as ‘Always effective’ (‘Wide Tube’ Mdn = 1, ‘Narrow Tube’ Mdn = 0.77; W=1, Z=-0.45, p = 1). Techniques involving the use of leafy sticks (which were pliable enough to be inserted into the tube in the ‘Narrow Tube’ phase) were classified as ‘Always effective’, and the success rate of ‘*leafy stick dip*’ did not change at the introduction of the ‘Narrow Tube’ (‘Wide Tube’ Mdn = 0.92, ‘Narrow Tube’ Mdn = 0.87; W=3, Z=-0.54, p=0.75).

An alternative coding scheme was considered, in which ‘*stick drop’* was classified along with *‘stick dip’* as ‘Partially effective’, due to the perceived difficulty of inserting rigid sticks into the Narrow Tube. This alternative coding scheme produced the following results. It should be noted that the pattern of the results remains the same – no individual used ‘Always effective’ techniques for more than 50% of attempts in the ‘Narrow Tube’ phase, but use of such techniques increased significantly in the ‘Narrow Tube’ phase.

**
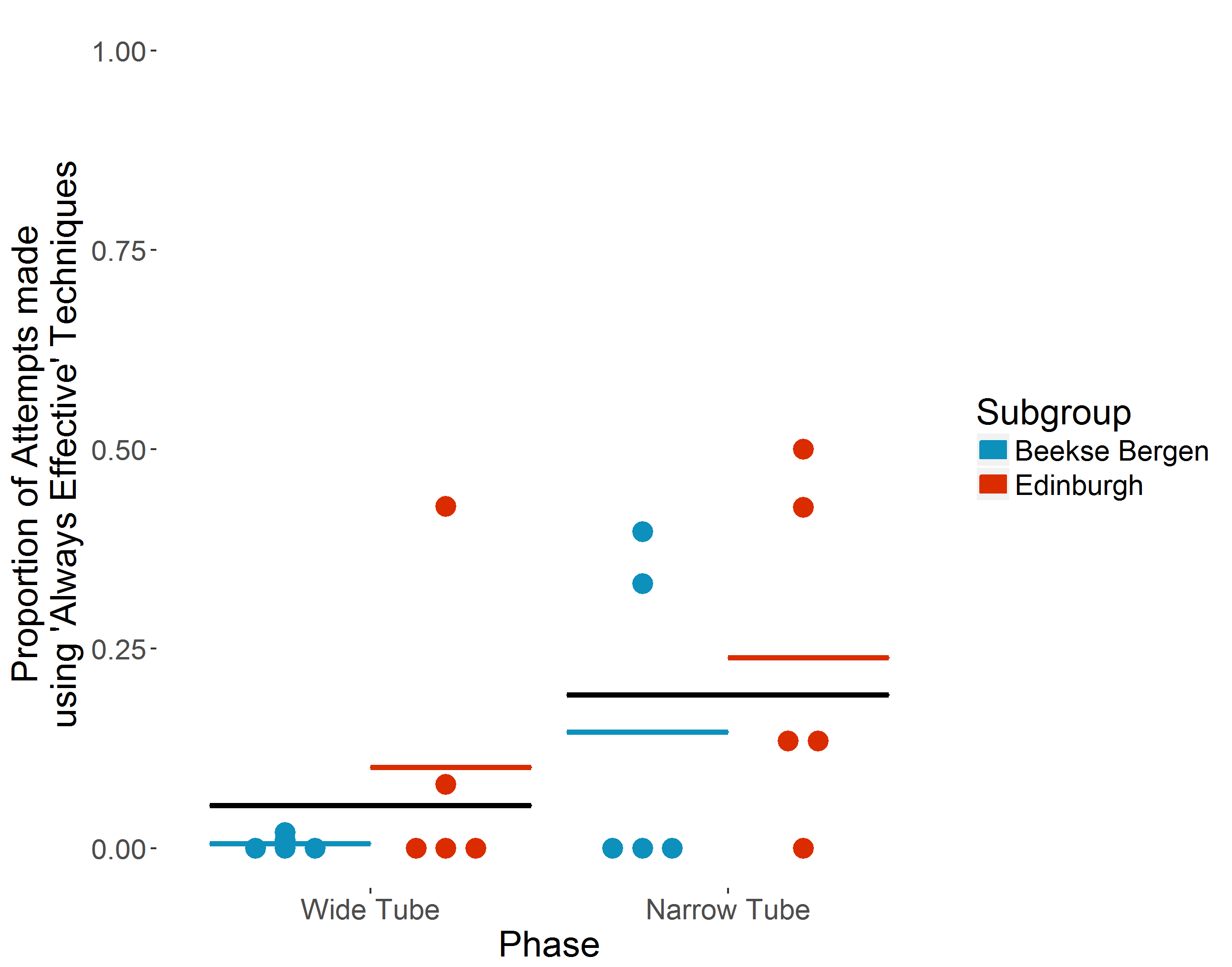
**

**Fig S1** Proportion of attempts made using ‘Always effective’ techniques in ‘Wide’ vs ‘Narrow’ tube phases by the ten chimpanzees that took part in both phases. Individual chimpanzees’ proportions are indicated by dots. Coloured horizontal bars show each subgroup’s mean proportion. Black horizontal bars show overall mean proportion.

The 15 individuals that interacted with the task used ‘Always effective’ techniques for 24 of 1548 attempts in the ‘Wide Tube’ phase, increasing to 422 of 1251 attempts in the ‘Narrow Tube’ phase. On average, each individual used ‘Always effective’ techniques for a mean of 3.8% (SD = 11.45, N=14) attempts in the ‘Wide Tube’ phase, increasing to a mean of 19.3% (SD = 20.16, N=11) of attempts in the ‘Narrow Tube’ phase (see Figure S1). No individual used ‘Always effective’ techniques for more than 50% of attempts in the ‘Narrow Tube’ condition.

As in the results presented in the main text, a binomial GLMM including Phase as a predictor and technique effectiveness as the outcome variable using this coding scheme indicates a significant increase in the use of ‘Always effective’ techniques in the ‘Narrow Tube’ phase.

The full model (including all 10 individuals that interacted with the task in both ‘Wide’ and ‘Narrow’ Tube phases) (see Table S1) indicates that use of ‘Always effective’ tool techniques increased significantly in the ‘Narrow Tube’ phase (*b* = 4.22, *p* = .0090). However it is important to note that while the model indicates significantly greater use of ‘Always effective’ techniques in the ‘Narrow Tube’ phase, the majority of individuals primarily used ‘Partially effective’ techniques in this phase.

Table S1.

*Results of full model GLMM on the effects of Phase upon ‘Always effective’ technique use. Variance, standard deviation, and correlation for the random intercept and slope for Individual by Phase is provided.*

|  | Estimate | [Wald 95% CI] | Std. Error | z value | P value |
| --- | --- | --- | --- | --- | --- |
| Intercept (including ‘Wide Tube’ phase) | -6.31 | [-9.65, -2.97] | 1.70 |  |  |
| Phase (‘Narrow Tube’) | 4.22 | [1.05, 7.39] | 1.62 | 2.611 | 0.00902** |
| Random effects | Variance | Std. Deviation | Correlation |  |  |
| Individual (intercept) | 12.52 | 3.54 |  |  |  |
| Slope by Phase | 8.01 | 2.83 | -0.86 |  |  |

**=*p*<0.01
